# Combined gas embolization and chemotherapy can result in complete tumor regression in a murine hepatocellular carcinoma model

**DOI:** 10.1063/5.0005329

**Published:** 2020-09-08

**Authors:** Jennifer N. Harmon, Foad Kabinejadian, Joseph L. Bull

**Affiliations:** Department of Biomedical Engineering, Tulane University, New Orleans, Louisiana 70118, USA

## Abstract

Hepatocellular carcinoma (HCC) is an intractable cancer with a high mortality rate. Transarterial chemoembolization (TACE), a non-curative method, is the first line therapy for intermediate stage patients. This effectively extends patient survival but requires a complicated intraarterial catheterization procedure and is poorly suited to repeated administration. Here, we investigate gas chemoembolization, a less invasive, more easily administered transient occlusion method that circumvents these limitations. We examined the efficacy of repeated embolization combined with systemically administered doxorubicin, the most common chemotherapeutic in TACE, or tirapazamine, a hypoxia-activated cytotoxic agent, in an ectopic xenograft model of HCC. Emboli were generated *in situ* using acoustic droplet vaporization, the noninvasive focused ultrasound-mediated conversion of intravenously administered perfluorocarbon microdroplets into microbubbles. Gas embolization alone significantly reduced the Ki67 index and tumor viability (11.6 ± 6.71% non-necrotic vs 100% in control; p < 0.01) after 3 treatments, as assessed by histological analysis. Mice treated for three weeks exhibited significant tumor regression compared to control (23.8 ± 5.37% of initial volume vs 427 ± 49.7% in controls, p < 0.01), irrespective of the chosen chemotherapeutic agent. However, an additional three weeks of monitoring post-treatment elucidated a significant difference in the tumor recurrence rate, with combined gas embolization and doxorubicin resulting in the best treatment outcomes (60% complete regression). While doxorubicin administration resulted in significant cardiotoxicity (p < 0.01), it strongly interacted with the droplet shells, reducing the systemic dose by 11.4%. Overall, gas chemoembolization shows promise as a developmental therapy and merits further study in more complex tumor models.

## INTRODUCTION

Hepatocellular carcinoma (HCC), the third leading cause of cancer-related death, is unresponsive to systemic chemotherapy alone.^1–4^ Due to comorbidities and generally late disease detection and diagnosis, the majority of HCC patients do not qualify for curative therapies (e.g., surgical resection or liver transplant).^3–5^ Transarterial chemoembolization (TACE) has been designated as the first line therapy for patients with Barcelona Clinic Liver Cancer (BCLC) stage B HCC.^6,7^ TACE uses an intraarterial catheter to locally deposit a chemotherapeutic and subsequently an embolic agent (e.g., polymer microspheres and foams) within vasculature upstream of a tumor to achieve selective ischemia and enhanced intratumoral retention of the chemotherapeutic. This procedure extends patient survival and is associated with few severe complications.^8,9^ However, the procedure is complicated, lacking in fine spatial resolution, and incompatible with frequent repeated administration, thereby limiting the ability to address any neovascularization following the initial embolization procedure.^10^ A less invasive, more spatially selective therapy with a less complex method of administration may allow for better lesion coverage and facilitate repeated treatments, resulting in more complete embolization and improved patient outcomes.

Gas embolization (GE) has been proposed as one such alternative.^11^ GE involves the use of noninvasive focused ultrasound (FUS) to selectively vaporize circulating perfluorocarbon droplets directly within the tumor vasculature, thereby generating localized gaseous occlusions.^12,13^ With intravenously injected or infused droplets, occlusions can rapidly be generated at multiple sites by moving the FUS focal spot rather than relying on precise placement of an intraarterial catheter. Extensive research has been conducted to characterize GE *in vitro* and *in silico*,^14–20^ culminating in recent *in vivo* experiments in which GE was proven to be effective at preventing disease progression in a murine tumor model.^12^

Incomplete tumor coverage was observed with the previous iteration of GE, however, likely resulting in a suboptimal tumor response.^12^ We hypothesized that the addition of a chemotherapeutic agent—either doxorubicin (DOX), the most commonly used agent in TACE procedures, or tirapazamine (TPZ), a hypoxia-activated drug that has shown promise when used alongside TACE in mouse models—would, in conjunction with modified treatment and acoustic parameters, result in more complete tumor coverage, with the intent of moving from a cessation of tumor growth to tumor regression.^21,22^ The current study initially investigates the effects of GE on tumor tissue during the early stages of treatment prior to assessing the therapeutic efficacy of gas chemoembolization (GCE) using DOX or TPZ as compared to GE alone, with respect to both tumor regression and risk of recurrence following the cessation of treatment.

## RESULTS

### Effects of GE in Early Stages of Treatment

**FIG. 1.**
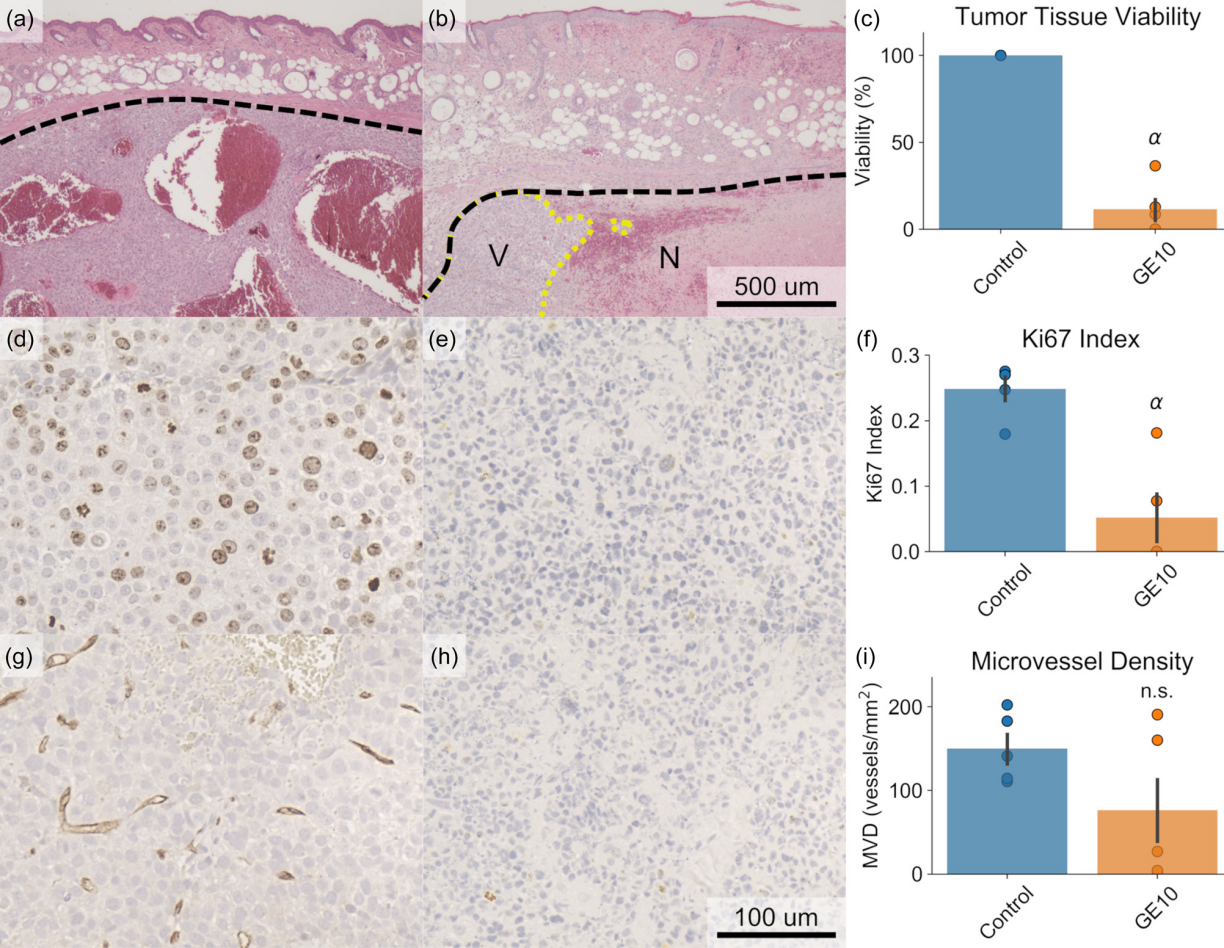
Histological analysis of tumor tissue after 10 days of treatment compared to an untreated control. (a)–(c) H&E staining was used to determine tumor viability. Widespread necrosis was observed in the treated tumors, resulting in a significant decrease in viability (α: p < 0.05 vs control, N = 5 per group). Tumor boundaries are marked in dashed black, whereas the boundary between necrotic and viable tumor tissue is marked in dotted yellow. V = viable; N = necrotic. (d)–(f) The Ki67 index was significantly reduced in treated tumors, indicating a reduced proliferative potential following treatment. (g)–(i) No significant difference was observed in microvessel density, as determined by CD31 staining. Representative example images from the control group (a), (d), and (g), in which tumors were harvested on day 1, and the treatment groups (b), (e), and (h), in which mice were treated with only gas embolization (GE) over 10 days prior to tumor harvest, are displayed for each type of stain.

The efficacy of GE in terms of its effect on tumor viability, proliferative potential, and microvessel density (MVD) was assessed during the early stages of treatment as compared to an untreated control. An ectopic xenograft model of HCC in mice was used as the model system. Treatment consisted of intravenous droplet administration and FUS exposure on a “1 day on, 3 days off” schedule for 10 days (i.e., 3 total treatments). Histological analysis was conducted on control (untreated) tumors harvested on day 1 and tumors treated with GE only (e.g., no chemotherapy) harvested on day 10 (GE10). It was determined that while treatment significantly reduced tumor viability [Figs. 1(a)–1(c), 11.6 ± 6.71% GE10 vs 100% control, p < 0.01] and the Ki67 index [Figs. 1(d)–1(f), 0.052 ± 0.036 GE10 vs 0.249 ± 0.018 control, p < 0.01], an analog for proliferation, the treatment had not significantly reduced the MVD in the remaining viable tumor tissue [Figs. 1(g)–1(I), p = 0.14]. Additionally, tumors in the treatment group had undergone significant regression compared to their initial volume by day 10 (p < 0.01). Overall, these results indicated that while the 10 day treatment course induced widespread necrosis and was effective in reducing the tumor volume, some amount of viable tumor tissue persisted and, due to the lack of significant reduction in MVD, was still perfused and capable of recurring following the cessation of treatment.

### Tumor Regression and Recurrence with GE and GCE

**FIG. 2.**
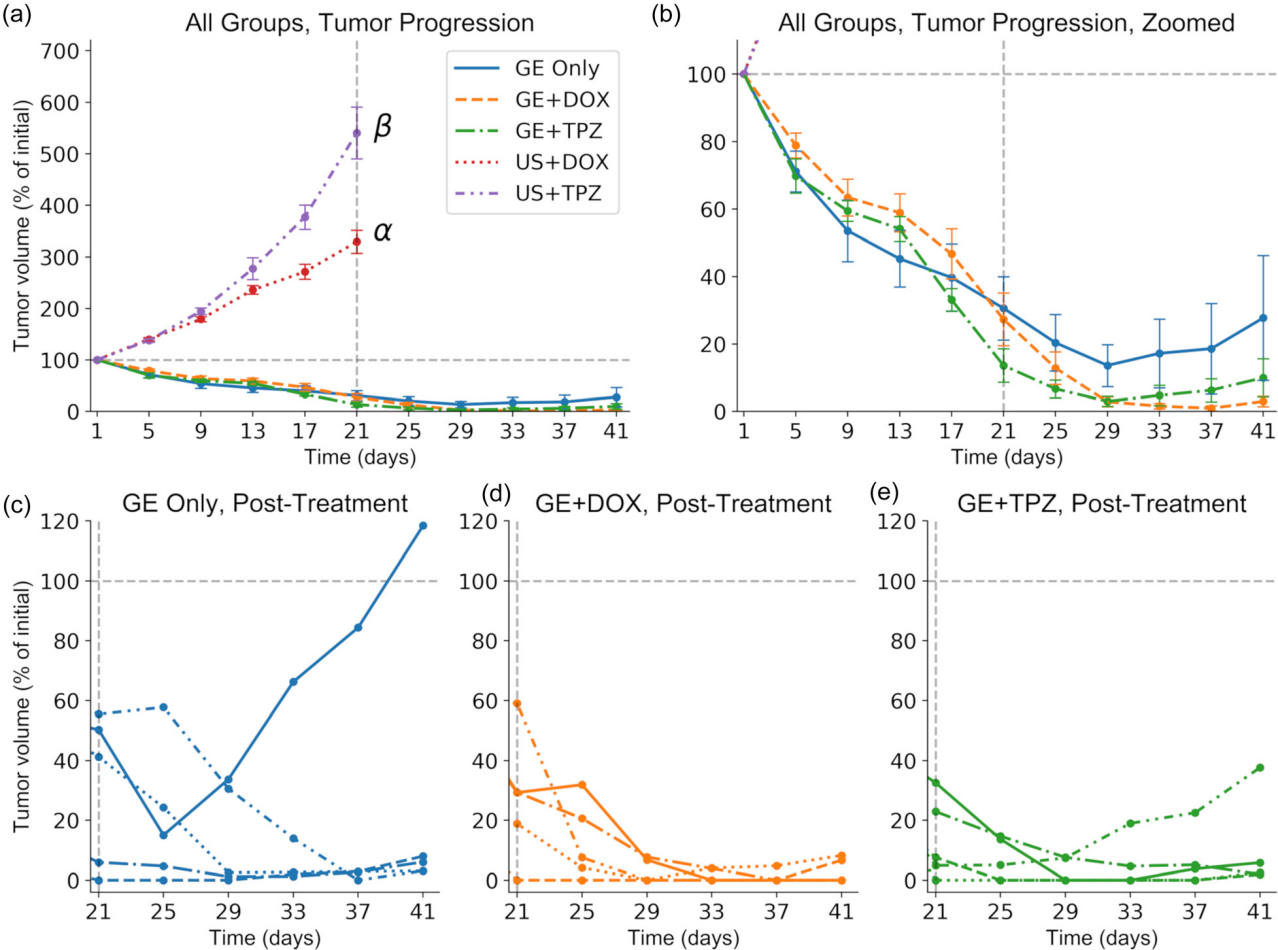
Gas chemoembolization induces substantial tumor regression, followed by recurrence in most cases. (a) The normalized tumor volume is plotted over time; ultrasound and chemotherapy only controls exhibited substantial tumor growth. Gray dashed lines denote the baseline volume (horizontal) and the final treatment day (vertical). α: p < 0.05 vs all treatment groups and ultrasound + tirapazamine (US+TPZ); β: p < 0.05 vs all treatment groups and ultrasound + doxorubicin (US+DOX). N = 5 per group. The significant difference observed between US+DOX and US+TPZ is likely due to severe toxicity and weight loss in the US+DOX group influencing tumor progression. (b) The plot is scaled to emphasize the three treatment groups. No significant differences in the final tumor volume were observed. (c)–(e) Normalized tumor volumes for each individual mouse within the three treatment groups are plotted following the cessation of treatment. Gas embolization (GE) Only and GE+TPZ exhibited a 100% tumor recurrence rate, whereas GE+DOX exhibited a 40% recurrence rate.

Following confirmation of the ability of GE alone to reduce the tumor viability and Ki67 index in the early stages of the treatment, the therapeutic efficacy of GE only or GCE (either GE+DOX or GE+TPZ) as compared to controls (ultrasound (US)+DOX or US+TPZ, without droplets) was investigated throughout a full treatment course. Treatments were administered on a 1 day on, 3 days off schedule for 21 days (i.e., 6 total treatments), followed by an additional 21 days of monitoring for tumor recurrence. The tumor volume, measured using calipers, was selected as the primary quantitative indicator of therapeutic efficacy during the treatment course, with additional qualitative monitoring using B-mode US imaging. Figure 2 illustrates the change in the tumor volume during and after treatment. All treatment groups exhibited significant tumor regression as compared to the US+DOX and US+TPZ control [p < 0.01, Fig. 2(a)]. The therapeutic outcomes were also an improvement over our previous study (i.e., tumor regression as compared to a cessation of growth), likely due to the increased insonation times during the first three treatments and increased droplet dosing in the current study.^12^ No significant differences in the tumor volume on day 21 were observed between the GE Only (30.6 ± 9.42% of initial volume, day 21), GE+DOX (27.3 ± 7.82%), and GE+TPZ (13.6 ± 4.97%) groups [p = 0.42, Fig. 2(b)], indicating that the addition of chemotherapy did not influence the rate of tumor volume reduction.

**FIG. 3.**
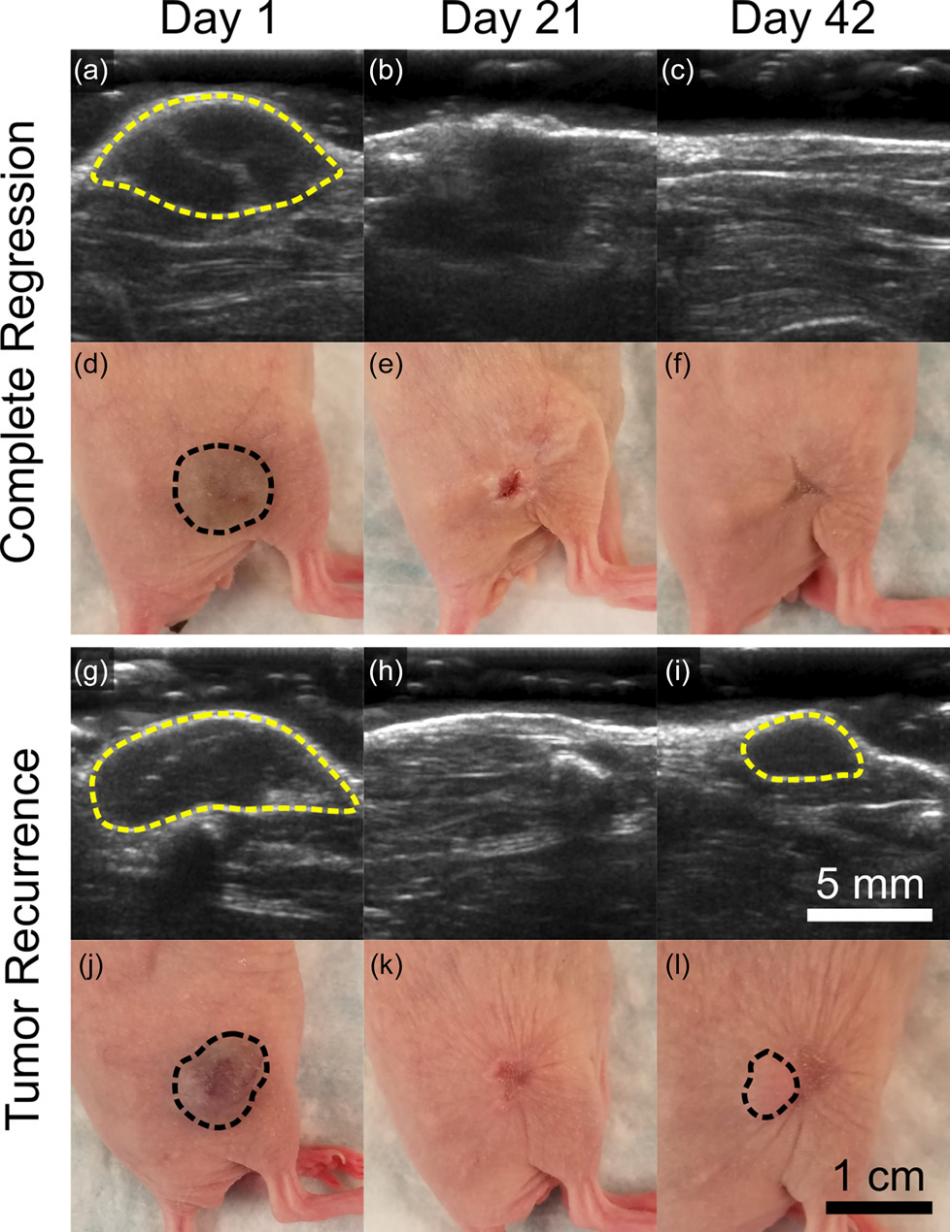
Ultrasound images and photos illustrating cases of complete regression and tumor recurrence. Tumors are outlined in dashed yellow in B-mode images [(a)–(c) and (g)–(i)] and dashed black in photos [(d)–(f) and (j)–(l)]. Mice were treated from day 1 through day 21 and subsequently monitored for tumor recurrence through day 42. The complete regression example (a)–(f) is from the gas embolization (GE) + doxorubicin (DOX) group, whereas the recurrence example (g)–(l) is from the GE Only group. Cases of complete regression were later confirmed using histological analysis.

All samples from each treatment group are plotted from day 21 to day 42, post-treatment, to illustrate cases of tumor recurrence in terms of tumor volume [Figs. 2(c)–2(e)]. Representative photographs and B-mode images of partial and complete regression cases (i.e., tumor recurrence or no recurrence) are displayed in Fig. 3. Mice were sacrificed on day 42, and tissue was collected to more accurately quantify the complete regression rate; complete regression was defined as the absence of identifiable tumor tissue on day 42 in H&E stained tissue sections. While no significant differences in the final tumor volume were observed between the GE Only (27.7 ± 18.5%), GE+DOX (2.97 ± 1.50%), and GE+TPZ (9.96 ± 5.68%) groups (p = 0.45, day 42), the complete regression rate was significantly higher in the GE+DOX group (60% vs 0% in the GE Only and GE+TPZ groups, p = 0.04). These results indicated that GE+DOX produced more favorable therapeutic outcomes as compared to GE Only and GE+TPZ, likely as a result of enhanced tumor coverage. The entirety of the lesion was embolized, exposed to DOX, or both, resulting in the observed enhanced complete regression rate. This was also likely responsible for the lack of a synergistic response in the GE+TPZ group; TPZ was activated only in those regions that had already been embolized and had no cytotoxic effect in the remaining viable regions of the tumor. Occlusion alone was sufficient for inducing necrosis; any activated TPZ present in the occluded regions did not add a detectable therapeutic benefit.

### Recurrent Tumors are Histologically Similar to Controls

**FIG. 4.**
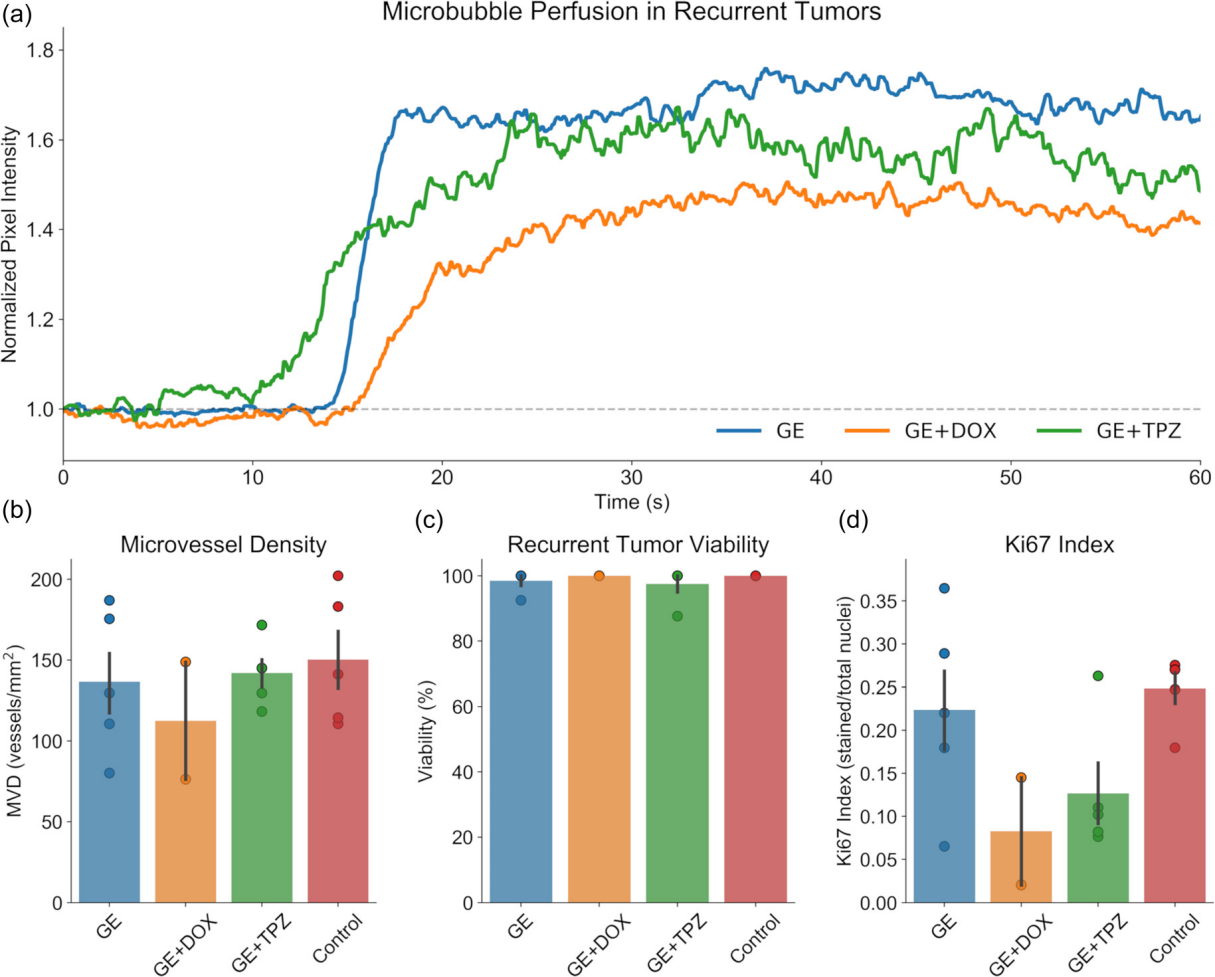
Recurrent tumors exhibit similar properties to control tumors. (a) Contrast-enhanced ultrasound imaging was conducted to visualize tumor perfusion *in vivo* prior to sacrifice. Perfusion was observed in recurrent tumors from each treatment group. (b)–(d) Histological analysis was conducted to examine the microvessel density [(b); CD31], tumor viability [(c); H&E], and proliferative potential [(d); Ki67] of the recurrent tumors. No significant differences were observed between recurrent tumors and untreated controls harvested on day 1. N = 5 per group, excepting gas embolization + doxorubicin (GE+DOX), which only exhibited tumor recurrence in 2/5 cases; in order to examine only the properties of the recurrent tumors, the additional 3 mice in which staining could not be conducted were excluded from these analyses.

On day 42, mice were imaged using contrast enhanced ultrasound imaging (CEUS) to determine if any recurrent tumor tissue was well-perfused or to provide further evidence indicating that the tumor had not recurred. Given that ADV and GE have been shown to induce some hemorrhage and vasoconstriction,^12^ it is possible that GE may have achieved a sublethal therapeutic benefit in the recurrent tumors with regard to perfusion or vascularization. It was determined that this was not the case; recurrent tumors in each treatment group were perfused [Fig. 4(a)]. Histological analysis was then conducted on recurrent tumors or tissue collected from the area in which the tumor had been prior to treatment, to determine if GE or GCE had a sustained impact on tumor viability, proliferation, and vascularity and to confirm complete regression in cases where recurrent tumors were not detected with ultrasound. Recurrent tumors exhibited no significant differences in MVD, necrosis, or Ki67 index as compared to an untreated control [Figs. 4(b)–4(d)], indicating that they were capable of continued growth.

### Doxorubicin-Induced Cardiotoxicity

**FIG. 5.**
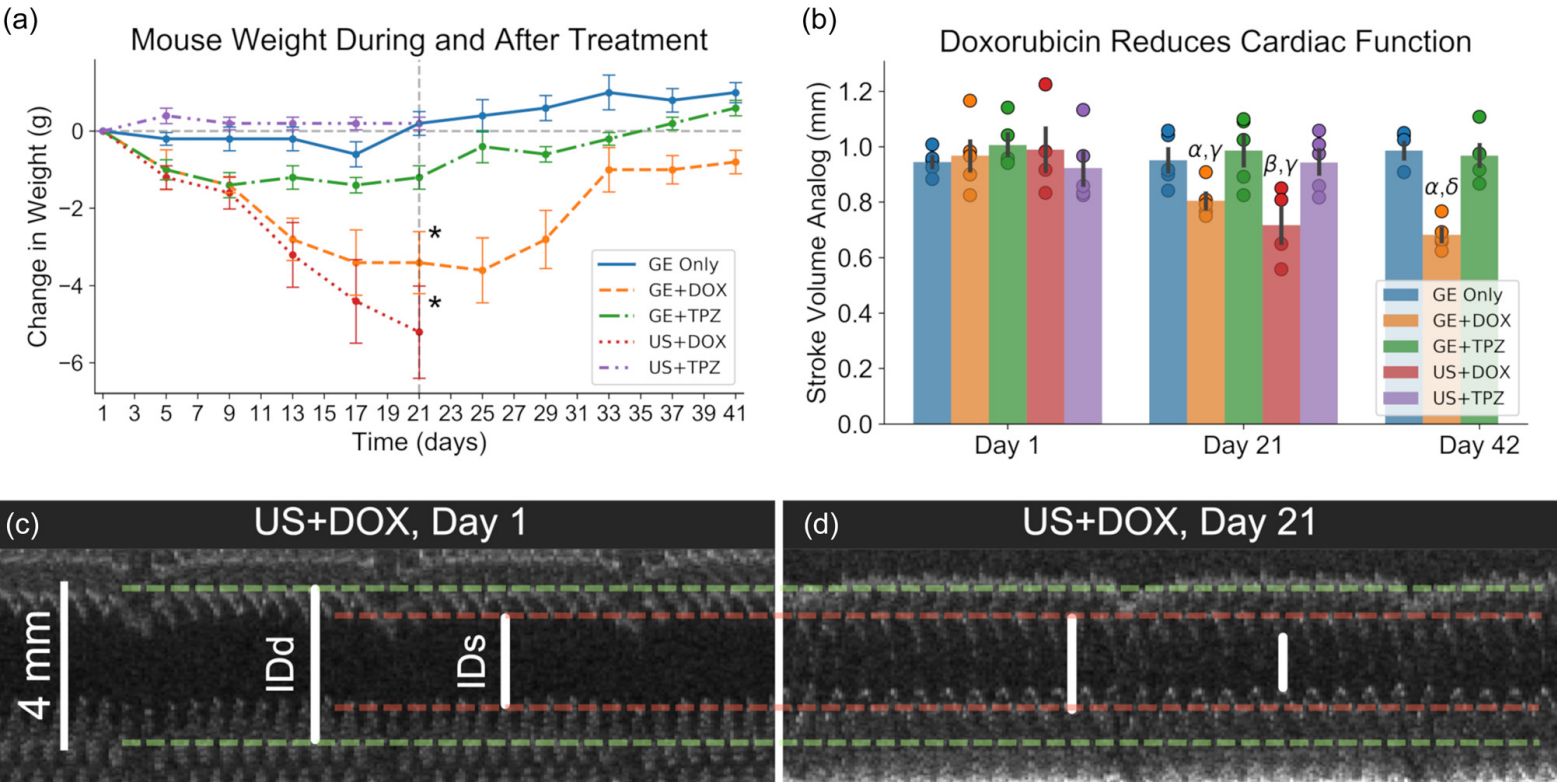
Significant cardiotoxic effects are observed in mice treated with doxorubicin. (a) Both doxorubicin (DOX) exposed groups exhibited significantly lower body weights following three weeks of treatment. *: p < 0.05 vs all non-DOX groups (N = 5 per group, US+DOX: N = 4). (b) Echocardiography indicated that both DOX groups exhibited significantly reduced cardiac function following three weeks of therapy. While the body weight in the GE+DOX group had recovered by day 21, cardiac function was still significantly reduced on day 42. α: p < 0.05 vs GE+DOX day 1; β: p < 0.05 vs ultrasound (US) + DOX day 1; γ: p < 0.05 vs GE Only, GE+TPZ, US+TPZ day 21; δ: p < 0.05 vs GE Only and GE+TPZ day 42. (c) and (d) Representative examples of M-mode ultrasound images on day 1 [(c); 0.83 mm SVA] and day 21 [(d); 0.65 mm SVA] are displayed. Clear reductions in both the inner left ventricular diameter at end diastole (IDd) and end systole (IDs) were observed.

While GE+DOX resulted in the most promising treatment outcomes, repeated systemic administration of DOX resulted in significant cardiotoxicity as compared to non-DOX groups. Although DOX-induced cardiotoxicity has been widely documented in the literature, the authors have chosen to include these results in order to elucidate the severity of these effects using the specific drug doses and the administration scheme utilized in this study. The cardiac function was assessed using a stroke volume analog (SVA); M-mode ultrasound imaging was used to measure the inner diameter of the left ventricle at end systole and at end diastole for quantification. The body weight [Fig. 5(a)] and cardiac function [Fig. 5(b)] were significantly reduced on day 21 in both the US+DOX (–5.2 ± 1.3 g; 0.72 ± 0.03 mm) and GE+DOX (–3.4 ± 0.9 g; 0.80 ± 0.01 mm) groups as compared to the GE Only (0.2 ± 0.3 g; 0.95 ± 0.02 mm), GE+TPZ (–1.2 ± 0.3 g; 0.97 ± 0.02 mm), and US+TPZ (0.2 ± 0.2 g; 0.94 ± 0.02 mm) groups (p < 0.01 for both the body weight and SVA). Example images collected using echocardiography demonstrating the impact of DOX administration are shown in Figs. 5(c) and 5(d). While the body weight had recovered by day 42, the cardiac function had not.

### DOX Binds to Droplet Shells

**FIG. 6.**
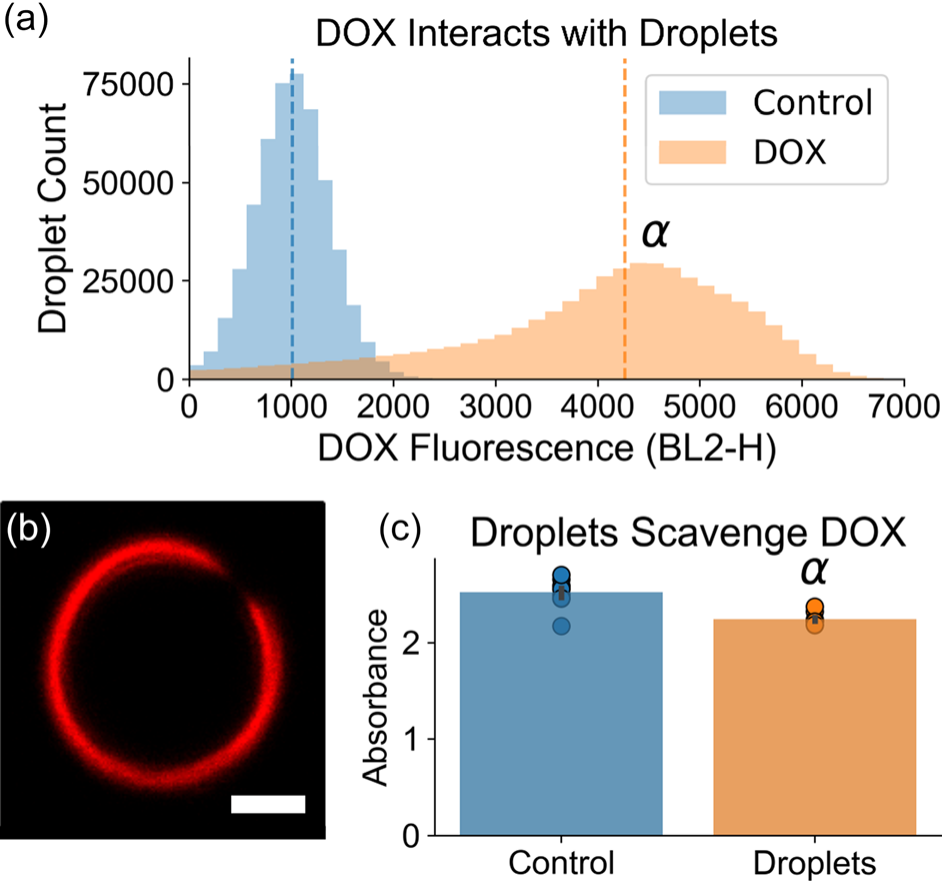
Doxorubicin (DOX) localizes to the droplet shell. (a) Flow cytometry analysis elucidated a significant increase in median fluorescence intensity in droplets that had been incubated with DOX, indicating that DOX was strongly interacting with the droplets. α: p < 0.05, DOX vs control. (b) Confocal microscopy confirmed the localization of DOX to the lipid droplet shell. Scale bar = 2 *μ*m. (C) The absorbance of the DOX solutions following incubation with the droplets was quantified as an analog to the DOX concentration. It was determined that the mean absorbance was significantly lower than controls (α: p < 0.05, DOX vs control; N = 10 per group). Droplets scavenged 11.4% of the total DOX dose.

A potential interaction between the DOX and droplets was investigated to determine if the droplets may serve as suitable local drug delivery vehicles in future work, in order to mitigate the observed DOX-induced cardiotoxicity. During treatment, droplets had initially been added to DOX solutions up to 15 min prior to administration. To match these conditions, droplets were incubated in either PBS (control) or a 0.714 mg/ml solution of DOX followed by two PBS washes and were subsequently analyzed using a flow cytometer. Droplets incubated with DOX exhibited a significantly increased median fluorescence intensity (2400 ± 163 DOX vs 355 ± 3.60 control, N = 10, p < 0.05), indicating that DOX was strongly interacting with the droplets [Fig. 6(a)]. Confocal microscopy confirmed the localization of DOX to the lipid droplet shell [Fig. 6(b)]. An absorbance assay indicated that in total, droplets sequestered 11.4% of the DOX dose to be administered to the GE+DOX group on each treatment day [Fig. 6(c); p < 0.05]. Though assays investigating potential release of DOX from the droplet shell in the presence of serum or whole blood must be conducted in future work, these initial results indicate that DOX localization to the tumor site using targeted droplets is a feasible approach. Future studies will investigate the safety and efficacy of fully localized drug delivery.

## DISCUSSION

The results presented here indicate that both GE and GCE are effective in significantly reducing tumor burden over a treatment course, with the additional benefit of suppression of tumor recurrence when combined with systemic doxorubicin in the GCE group. These results are a significant improvement over our previous study, in which we achieved a cessation of tumor growth but did not induce regression.^12^ Additionally, these results provide insight into the behavior of any remaining tumor tissue following the cessation of therapy. No significant differences were observed between recurrent and control tumors, suggesting that either a combination therapy (e.g., GE+DOX) or adjuvant therapy following GE—potentially including radio frequency or FUS-based thermal ablation, or surgical resection of remaining tumor tissue following GE downstaging—will be necessary to ensure sustained disease-free survival in future studies.^23,24^

One limitation of the current method is the systemic chemotherapy administration. TPZ proved to be ineffective at enhancing the therapeutic benefit conferred by GE, likely due to its activation solely in embolized regions in which occlusion alone was already sufficient to induce necrosis. DOX, however, did enhance the therapeutic effect of GE, but exhibited severe cardiotoxicity. Considering the localized delivery and enhanced intratumoral retention of chemotherapeutics when using TACE, the current standard for embolization of HCC, it is clear that purely systemic drug delivery will prove unacceptable in future iterations of the therapy. GE lends itself well to localized drug delivery, as evidenced by the reported loading of DOX onto the droplet shell. Drug loading onto the shell and into the core of these droplets, as well as efficient drug release following ADV, has been thoroughly explored and is well-documented in the literature; these techniques will be employed in the future to maintain the enhanced therapeutic efficacy achieved with GCE while minimizing cardiotoxic effects.^25–27^ Selective local drug release concomitant with gas embolization will facilitate retention of the chemotherapeutic agents within the tumor and will limit the amount of freely circulating drug. Alongside the development of localized drug delivery, future studies will utilize image-guided FUS to ensure localization of ADV to the lesion site and to assist with tumor detection for the treatment of *in situ* tumors. We have recently developed a prototype for ultrasound guided gas embolization using a single, widely available linear array transducer, with the intention of maximizing the ease of treatment administration and accessibility.^28^

Overall, GCE shows promise as an alternative therapy to TACE given its ability to consistently induce significant tumor regression and the ease of use and widespread accessibility of ultrasound. Moving forward, experiments in larger animals and more complex, *in situ* tumor models will be necessary to confirm the scalability of GCE and its efficacy in more physiologically relevant scenarios.

## METHODS

### Droplet and Microbubble Fabrication

Droplets targeted to the integrin α_v_β_3_, an angiogenic marker overexpressed in HCC, were fabricated as described previously.^12^ Microbubbles were produced as follows: 1,2-distearoyl-sn-glycero-3-phosphocholine (90 mol%, DSPC, Avanti Polar Lipids, Alabaster, AL, USA) and 1,2-distearoyl-sn-glycero-3-phosphoethanolamine-N-[methoxy(polyethylene glycol)-2000] (ammonium salt) (10 mol%, DSPE-mPEG2000, Avanti Polar Lipids) dissolved in chloroform were mixed and subsequently dried under vacuum to produce a lipid thin film. The film was hydrated using a mixture of phosphate buffered saline (PBS, 80% v/v), propylene glycol (10% v/v), and glycerol (10% v/v) and heated to 70 °C while stirring to produce a lipid blend, which was then added to an amber glass vial. The headspace was filled with perfluoropropane gas (Praxair, Danbury, CT, USA) prior to shaking for 45 s. The resulting microbubble suspension was characterized using a Coulter Counter (Multisizer 4e, Beckman-Coulter, Brea, CA, USA).

### Cell Culture and Tumor Model

Hep3B cells obtained from the American Type Culture Collection (RRID:CVCL_0326, ATCC, Manassas, VA, USA) were cultured at 37 °C in 5% carbon dioxide using media consisting of DMEM (Gibco, Waltham, MA, USA) supplemented with 10% fetal bovine serum (FBS, Gibco) and 1% penicillin-streptomycin (Gibco). The cell line was characterized using STR profiling by ATCC. Hairless severe combined immunodeficiency (SCID) mice (6–8 weeks old, Jackson Laboratory, Bar Harbor, ME, USA) were subjected to subcutaneous injections of 5 million cells in a 1:1 mixture of media to Matrigel (Corning Inc., Corning, NY, USA), administered on the right flank. All animal procedures were conducted with the approval of the Institutional Animal Care and Use Committee (IACUC) at Tulane University (Protocol Approval number 4442).

### Therapy and Tumor Monitoring

Tumor progression was monitored for 4–6 weeks using calipers and B-mode ultrasound imaging prior to assigning mice to one of the following groups: (i) GE only; (ii) GE+TPZ; (iii) GE+DOX; (iv) US+TPZ; (v) US+DOX; (vi) GE only, 10-day treatment course (GE10); and (vii) untreated control. Mice were assigned such that the initial volume (½ width^2^ * length) did not vary significantly between groups (ANOVA, N = 5 per group, p = 0.35; overall average 234 ± 1.13 mm^3^).^29^ Mice assigned to group (vi) or (vii) were sacrificed on either day 10 or day 1, respectively, to collect tumor tissue for histological analysis. Otherwise, treatment proceeded as described below.

Mice were treated on a 1 day on 3 days off schedule for 21 days, resulting in 6 total treatments. The procedure consisted of administering either no injection [groups (i), (iii), (v), and (vi)] or an intraperitoneal injection of TPZ in 20% v/v dimethyl sulfoxide (DMSO) in PBS [20 mg/kg, 150 *μ*l; groups (ii), (iv)]. After 10 min, either a droplet suspension in PBS [1 × 10^9^ droplets, 125 *μ*l; groups (i)–(iii) and (vi)] or PBS alone [groups (iv) and (v)] was injected through the tail vein containing no additional agent [groups (i), (ii), (iv), and (vi)] or DOX [4 mg/kg; groups (iii) and (v)]. Mice were then anesthetized using isoflurane (4% induction, 1.5–2% maintenance, 1 L/min O_2_) and weighed, and a photograph of the tumor was captured. Acoustic coupling gel was applied to the tumor prior to exposing the lesion to continuous pulsed FUS operated at 2.5 MHz (13 cycle pulses, 100 Hz pulse repetition frequency, 5.34 MPa peak negative pressure) using a focused single element transducer (H-108, Sonic Concepts, Bothell, WA, USA) equipped with a polycarbonate coupling cone. The transducer was manually scanned over the tumor tissue for 5 min for the first three treatments and 2 minutes for the latter three, in response to the observed reduction in the tumor volume in the treatment groups. Following treatment, B-mode ultrasound images of the tumor were captured, a photograph of the lesion was taken, and the mouse was observed until awake and ambulatory.

Following treatment, mice were monitored for an additional 21 days, excluding the US+TPZ and US+DOX groups, as the tumor size had approached the maximum allowable by the approved IACUC protocol by day 21, thereby requiring euthanasia. The body weight, photographs, B-mode images, and caliper measurements were collected to monitor for recovery from chemo-induced weight loss, if any had been observed, and tumor recurrence.

### Contrast Enhanced Ultrasound Imaging

On the final day of monitoring, prior to sacrifice, CEUS was conducted to assess perfusion in recurrent tumors. Tumors were imaged using a contrast-specific imaging mode operated on a Verasonics Vantage Research Ultrasound system (256 channel, high frequency configuration, Verasonics Inc., Kirkland, WA, USA) and a linear array transducer operated at 15.625 MHz (L22–14v, Verasonics). An IV injection of microbubble contrast agent (1 × 10^9^, in 100 *μ*l of PBS) was administered 15 s after initiating imaging. Imaging continued for 60 s after visualizing wash-in of the contrast agent into the tumor vasculature, and a cine loop was saved for analysis. The average pixel intensity within the tumor was quantified using a custom MATLAB script (MathWorks Inc., Natick, MA, USA) and plotted over time.

### Echocardiography

Cardiac function was assessed on Days 1, 21, and 42 using echocardiography. Mice were anesthetized using isoflurane as described previously. The transducer was aligned perpendicular to the long axis of the heart to capture a cross section of the left ventricle. Imaging was conducted at 60 frames per second; 4 s duration cine loops were captured for analysis. A custom MATLAB script generated a simulated M-mode image from each cine loop and allowed users to identify the inner left ventricular diameter at end diastole (IDd) and end systole (IDs), averaged across three distinct cardiac cycles. Cardiac function was condensed into a single continuous stroke volume analog (SVA), accounting for the 2-dimensional nature of the data. The formula SVA = IDd—IDs was used for quantification.

### Histology

Recurrent tumors, or skin and muscle from the former tumor site in cases of complete regression, were collected following sacrifice. Tissue was fixed in 10% formalin (Sigma Aldrich), processed and embedded in paraffin, and sectioned at a thickness of 5 *μ*m. Sections were either stained with hematoxylin and eosin (H&E) or subjected to immunohistochemical staining for CD31 (ab28364, Abcam, Cambridge, UK) or Ki67 (ab15580, Abcam) using a previously described procedure.^12^ The MVD, Ki67 index, and tumor viability were quantified as described previously, except that 3 “hot spots” imaged at 20× magnification were analyzed per independent sample.^12^ Complete regression was defined as an absence of identifiable tumor tissue in the H&E stained sections of tissue collected on day 42, following the full treatment course and monitoring period.

### Assessment of DOX-Droplet Interaction

Droplets (1 × 10^9^) were incubated in either a 0.71 mg/ml solution of DOX in PBS (experimental) or PBS only (control) for 15 min prior to centrifugation. The supernatant was collected for further analysis, and the droplet pellet was washed twice with PBS via repeated resuspension and centrifugation. Droplets were then resuspended in 400 *μ*l of PBS and analyzed using an acoustic focusing flow cytometer (Attune Acoustic Focusing Cytometer, Applied Biosystems, Foster City, CA, USA) equipped with a 488 nm excitation laser and a 574/26 nm emission filter. Detector gain values were selected, and binary gating was conducted such that unstained samples fell below 10^3^ arbitrary units (AU) and stained samples fell within the linear range of the detector (10^4^–10^5^ AU). A total of 500 000 events were analyzed per sample. Data were collected—with identical hardware and software settings for all samples—using Attune cytometric software (v2.1.0, Applied Biosystems) and quantified using Python (v3.6.5) and the FlowCytometryTools package (v0.5.0). Matplotlib (v3.1.1) was used to generate histograms with linearly scaled x-axes for visualization.^30^ Droplets were subsequently analyzed using an inverted point scanning confocal microscope (Eclipse Ti2, Nikon, Minato, Tokyo, Japan). Images were captured at 900 × magnification (60 × oil immersion objective, 15 × optical zoom, Nikon) using a 488 nm excitation laser coupled with a 595/50 nm emission filter. Images were processed for display using NIS Elements software (Nikon).

The supernatant was subjected to an absorbance assay using a SpectraMax i3× plate reader (480 nm, Molecular Devices, San Jose, CA, USA) alongside a logarithmic standard curve consisting of 1 × 10^−3^–1 mg/ml DOX. Control samples, in which DOX solutions were prepared but not incubated with droplets, were also analyzed for comparison.

### Statistical Analysis

All statistical analyses were conducted using Python (v3.6.5) with the Scipy stats (v1.3.2) and statsmodels (v0.9.0) packages.^31–33^ All visualizations were generated using Matplotlib and Seaborn (v0.9.0). α = 0.05 for all analyses. All tests are two-sided. Data are presented and plotted as *μ* ± SEM. One-way ANOVA analyses were used to compare tumor volumes on day 21 and day 42; histological data between treatment groups and the control (MVD, Ki67 index, viability); and the change in the body weight and SVA between groups on day 21 and 42 and within groups between day 1, day 21, and day 42. P-values were adjusted for multiple comparisons. Independent t-tests were used to compare histological data between GE10 and the control, as well as the flow cytometry (median fluorescence intensity) and absorbance assay results. N = 5 for all mouse experiments and N = 10 for the DOX-droplet interaction assessment unless otherwise specified.

## Data Availability

The data that support the findings of this study are available from the corresponding author upon reasonable request.
